# NEDD4L in human tumors: regulatory mechanisms and dual effects on anti-tumor and pro-tumor

**DOI:** 10.3389/fphar.2023.1291773

**Published:** 2023-11-09

**Authors:** Meng Zhang, Zhenyong Zhang, Xin Tian, Enchong Zhang, Yichun Wang, Jun Tang, Jianzhu Zhao

**Affiliations:** ^1^ Department of Oncology, Shengjing Hospital of China Medical University, Shenyang, China; ^2^ Department of Urology, Shengjing Hospital of China Medical University, Shenyang, China; ^3^ Department of Thoracic Surgery, Shengjing Hospital of China Medical University, Shenyang, China

**Keywords:** NEDD4L, ubiquitination, E3 ubiquitin ligase, cancer, signaling pathways, molecular mechanisms, targeted drugs

## Abstract

Tumorigenesis and tumor development are closely related to the abnormal regulation of ubiquitination. Neural precursor cell expressed developmentally downregulated 4-like (NEDD4L), an E3 ubiquitin ligase critical to the ubiquitination process, plays key roles in the regulation of cancer stem cells, as well as tumor cell functions, including cell proliferation, apoptosis, cell cycle regulation, migration, invasion, epithelial–mesenchymal transition (EMT), and tumor drug resistance, by controlling subsequent protein degradation through ubiquitination. NEDD4L primarily functions as a tumor suppressor in several tumors but also plays an oncogenic role in certain tumors. In this review, we comprehensively summarize the relevant signaling pathways of NEDD4L in tumors, the regulatory mechanisms of its upstream regulatory molecules and downstream substrates, and the resulting functional alterations. Overall, therapeutic strategies targeting NEDD4L to treat cancer may be feasible.

## 1 Introduction

Ubiquitination, an important post-translational modification of proteins, is involved in the regulation of various physiological functions in organisms (Buetow and Huang, 2016). Ubiquitination is a multistage enzymatic reaction mediated mainly by ubiquitin-activating enzymes (E1s), ubiquitin-conjugating enzymes (E2s), and ubiquitin ligases (E3s) (Hershko and Ciechanover, 1998). In this series of enzymatic cascade reactions, E3s play a key role in the specific recognition of target proteins and regulation of the ubiquitination system (Berndsen and Wolberger, 2014). Many studies have shown that the occurrence and development of tumors are closely related to abnormal ubiquitination (Mansour, 2018; Pérez-Benavente et al., 2020). Since E3s are known to play a pivotal role in the ubiquitination system and target protein recognition, they are primer drivers regulating tumor occurrence and development (Satija et al., 2013; Mansour, 2018).

Based on differences in the characteristic structural domains of E3s and the mechanism of ubiquitin delivery to target proteins, E3s can be classified into three family types: those containing the Really Interesting New Gene (RING)-finger structural domain, those containing the Homologous to the E6-AP Carboxyl Terminus (HECT) structural domain, and those containing the U-box structural domain (Wang et al., 2020). According to the structures of their N-terminal protein–protein interaction domains, the 28 human HECT E3 ligases are further divided into three classes: the NEDD4 family (9 members), the HRC family (6 members), and other HECTs (13 members) (Rotin and Kumar, 2009). Furthermore, the NEDD4 family includes 9 family members: NEDD4 (also referred to as NEDD4-1), NEDD4L (also referred to as NEDD4-2), ITCH, SMURF1, SMURF2, WWP1, WWP2, NEDL1, and NEDL2 (Zou et al., 2015). In this review, we focus on the structure and function of NEDD4L and the role of NEDD4L in malignant tumors.

**FIGURE 1:**
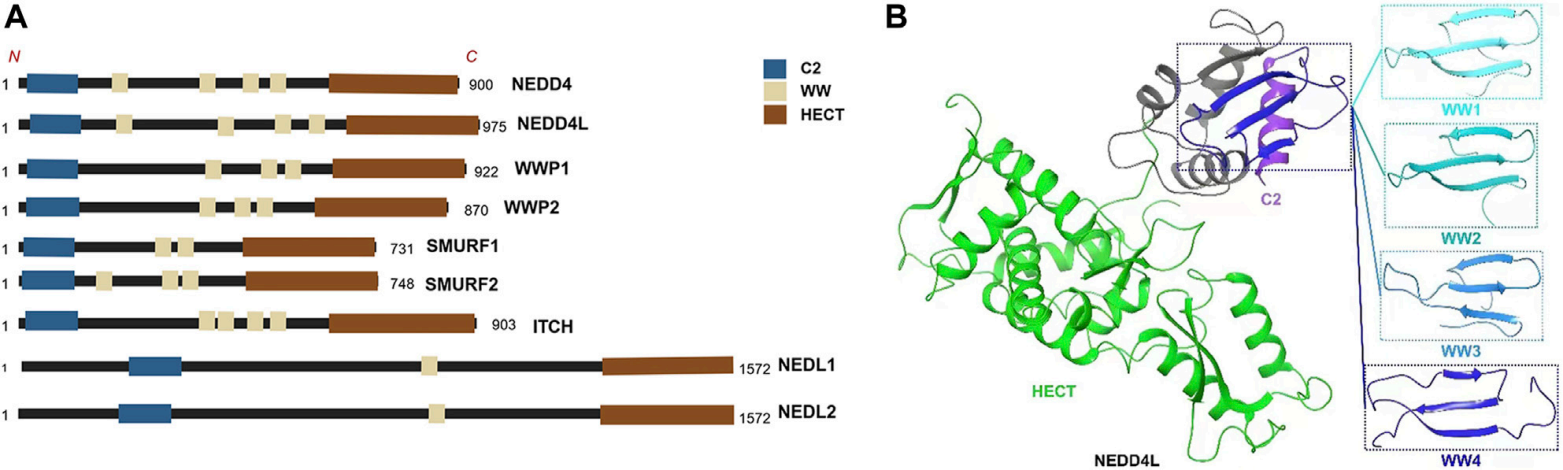
**(A)** The structures of all members of NEDD4 family. https://biorender.com **(B)** The3D structure of NEDD4L.

All members of the NEDD4 family have unique domain structures, each consisting of a C2 domain, 2–4 WW domains, and a HECT-type ligase domain (Zou et al., 2015) (Figure 1A). The C2 domain mediates the binding of NEDD4 members to cell membranes and participates in substrate recognition (Plant et al., 1997). The WW domains are named after their two tryptophan (tryptophan, W) residues and interact with the PY motif (proline, P; tyrosine, Y; PPYY) of the substrate protein or phosphor-serine/threonine residues (Staub et al., 1996; Ingham et al., 2005; Hatstat et al., 2021). The HECT domain is a conserved C-terminal catalytic domain with the enzymatic activity of E3 ligases inherent to the NEDD4 family (Scheffner and Kumar, 2014; Fajner et al., 2017). Although the E3 ligases of the NEDD4 family are structurally similar, they exert widely differing functions due to their different WW domains (Mari et al., 2014). NEDD4L is the closest homolog of family member NEDD4-1 (Yang and Kumar, 2010). The human NEDD4L gene is located on chromosome 18q21 and includes 38 exons that can generate multiple spliced mRNAs (Chen et al., 2001; Dunn et al., 2002; Araki et al., 2008). The modular structure of NEDD4L consists of 1 C2, 1 HECT, and 4 WW domains (Goel et al., 2015) (Figure 1B). NEDD4L is expressed in several tissues, especially the brain, heart, lungs, kidneys, and liver (Goel et al., 2015). NEDD4L has been reported to ubiquitinate substrates via Lys-6, Lys-11, Lys-48, Lys-63, Lys-27, and Lys-29 linkages and lead to degradation of substrate by lysosomes or the proteasome, and/or change downstream cell signaling pathways (Fotia et al., 2006; Ding et al., 2013). Despite being highly homologous to NEDD4-1, NEDD4L has a different substrate library (Harvey and Kumar, 1999; Yang and Kumar, 2010), implying that it may have features different from those of NEDD4-1. The earliest and most widely studied aspect of NEDD4L was its regulation of the epithelial Na^+^ channel (ENaC), which is associated with cardiovascular and kidney disease (Dinudom et al., 2004; Shi et al., 2008; Zhang D. D. et al., 2021). NEDD4L is also involved in antiviral immunity, ophthalmic diseases, and the inflammatory responses (Gao et al., 2021; Li H. et al., 2022; Santilli et al., 2022; Li et al., 2023).

**FIGURE 2:**
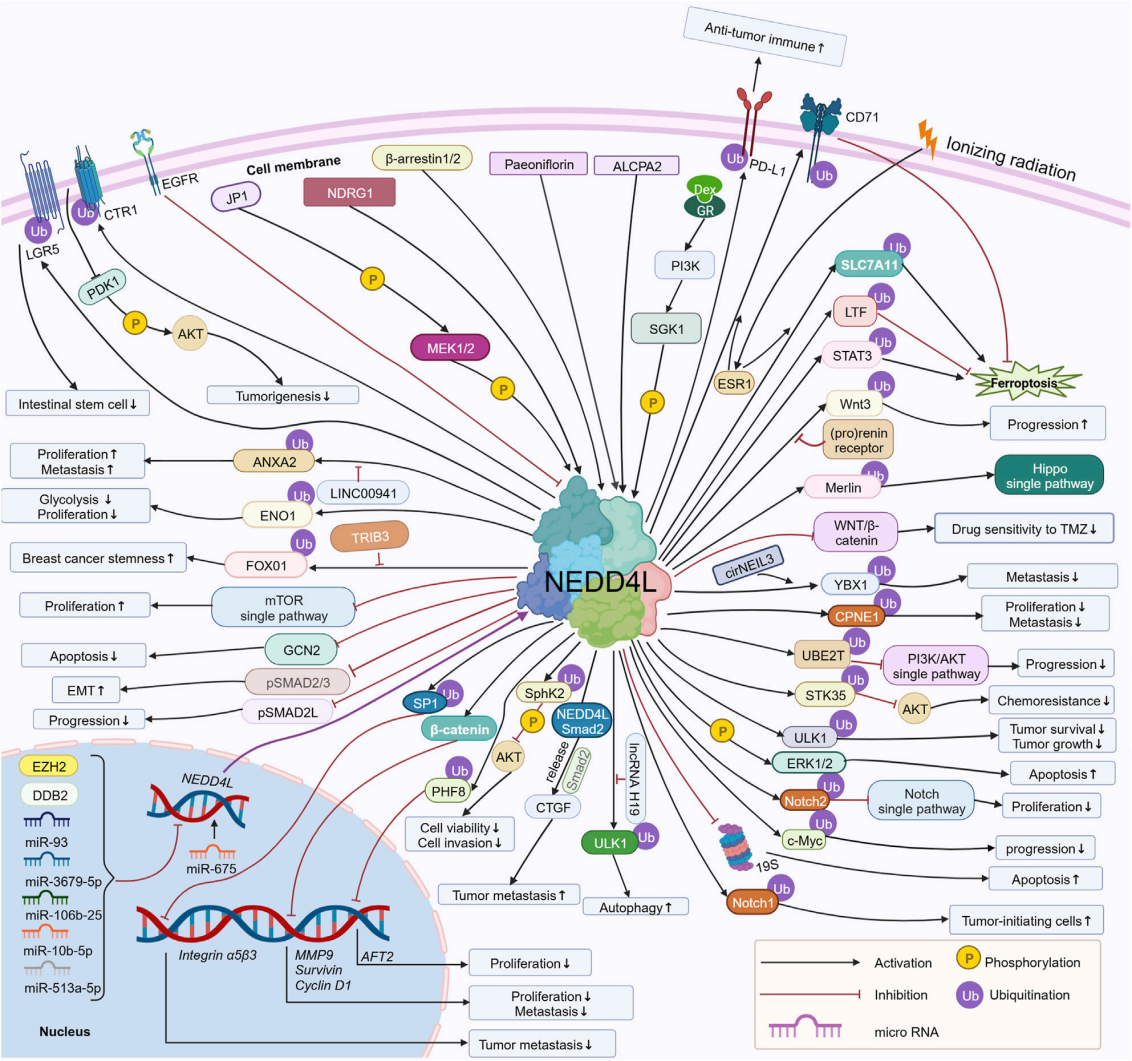
Regulatory mechanisms of NEDD4L involved in tumor cells. Upstream regulators of NEDD4L, including miR-93, miR-3679-5p, miR-106b-25, miR-10b-5p, miR-513a-5p, EGFR, DDB2, EZH2, SGK1, NDRG1, β-arrestin1/2, MEK1/2, ALCPA2, and Paeoniflorin, regulate NEDD4L via phosphorylation of NEDD4L, regulation of transcription, and others. NEDD4L regulates downstream substrates, including LGR5, ANXA2, ENO1, FOXO1, GCN2, pSMAD2/3, pSMAD2L, SP1, β-catenin, PHIF8, SphK2, ULK1, Notch1/2, c-Myc, ERK1/2, STK35, PI3KCA, UBE2T and CD71 via ubiquitination, phosphorylation, and the regulation of substrate transcription. The total network regulates the growth, proliferation, migration and invasion, apoptosis, autophagy, EMT, and drug sensitivity of tumor cells, as well as tumorigenesis and tumor progression. https://biorender.com.

Recently, it was reported that NEDD4L is aberrantly expressed in tumors and regulates tumorigenesis and tumor development (Xie et al., 2021). In 2021, Shang dan Xie et al. reviewed the potential role of NEDD4L in human cancers and the associated regulatory mechanism in a logical order of different tumors in *Frontiers in Oncology*. Since then, additional studies (approximately 30 articles) related to NEDD4L in tumors have been reported. In our review, we provide an updated scenario of recent studies on the role of NEDD4L in tumors, including non-solid tumors. The upstream molecules of NEDD4L regulate NEDD4L by regulating transcription, phosphorylation, non-coding RNA, etc. NEDD4L regulates downstream substrates through ubiquitination, regulation of transcription, phosphorylation, etc. Here, we describe the upstream regulatory molecules and downstream substrates of NEDD4, focusing on the signaling pathways associated with NEDD4L in tumors, the regulatory mechanisms of upstream regulatory molecules and downstream substrates, and the resulting functional alterations related to cell proliferation, apoptosis, cell cycle, migration, invasion, epithelial–mesenchymal transition (EMT), cancer stem cell behavior, and tumor drug resistance (Figure 2). Of note, the most important and extensive role NEDD4L plays in regulating tumor cell function is in ubiquitinating substrates. Finally, we discuss the potential of NEDD4L as a therapeutic target.

## 2 NEDD4L expression in human cancers

### 2.1 Low NEDD4L expression

Several studies have reported the low expression of NEDD4L in tumor tissues, including mRNA and protein expression, indicated its role as a tumor suppressor in specific types of cancer. Sakashita et al. (2013) revealed that the mRNA expression of *NEDD4L* was significantly lower in non-small cell lung cancer than in normal tissues, and the results were consistent in both squamous carcinoma and adenocarcinoma. In line with these findings, Li G. et al. (2022) analyzed public databases and observed that *NEDD4L* mRNA expression was lower in lung adenocarcinoma (LUAD) tissues than in paraneoplastic tissues. Moreover, Zhao et al. (2018) demonstrated that *NEDD4L* mRNA expression was downregulated in liver cancer tissues compared to that in paraneoplastic tissues. Notably, Zhao et al. (2021) found that *NEDD4L* was the only NEDD4 family member differentially expressed in renal cell carcinoma compared with levels in normal kidney tissues. Additionally, the transcript levels of *NEDD4L* were lower in clear-cell renal cell cancer tissues than in normal kidney tissues. Besides, Jiang et al. (2019) reported that the mRNA expression of *NEDD4L* was lower in gastric cancer tissues than in normal gastric tissues. Furthermore, Tanksley et al. (2013) found that *NEDD4L* mRNA was downregulated in all tumor stages of colorectal cancer. In addition, a bioinformatics analysis reported that *NEDD4L* mRNA expression was significantly downregulated in esophageal cancer compared to that in normal esophageal tissue (Zhang X. et al., 2021). On the other side, *NEDD4L* gene expression has also been reported in non-solid tumors. *NEDD4L* mRNA was expressed at low levels in acute myeloid leukemia (AML), and low expression was associated with a normal karyotype and with FLT3 and NPM1 mutations. Additionally, low expression positively correlated with complex karyotypes and TP53 mutations, suggesting that aberrant *NEDD4L* expression is associated with multiple genetic events in AML (Chu et al., 2021).

Similarly, low expression of NEDD4L protein has been reported in several tumors. Immunohistochemical staining was used to confirm that NEDD4L protein expression was downregulated in LUAD (Li G. et al., 2022), infiltrating ovarian epithelial tumor (Yang et al., 2015), hepatocellular carcinoma (Zhao et al., 2018), renal cell carcinoma (Zhao et al., 2021), rectal carcinoma (Tanksley et al., 2013), gastric cancer (Gao et al., 2012; Jiang et al., 2019), and glioma (He et al., 2012) compared to that in paraneoplastic or normal tissues. However, the expression level of the NEDD4L protein was not associated with any histopathological type of ovarian cancer (Yang et al., 2015). Besides, NEDD4L protein expression was downregulated in several different hepatocellular carcinoma cell lines compared to that in normal liver cell lines (Zhao et al., 2018). NEDD4L expression fluctuated from strong to weak as the pathological grade of gliomas increased (He et al., 2012). Additionally, NEDD4L was expressed at significantly lower levels in endometrial cancer tissues than in benign endometrial lesions (Yilmaz et al., 2018). However, whether the expressions are different between endometrial tumor and normal endometrial tissues is still unclear.

### 2.2 High NEDD4L expression

Despite its low expression in most tumors, NEDD4L is highly expressed in a few, such as Sézary syndrome (Booken et al., 2008), melanoma (Kito et al., 2014), and invasive gallbladder cancer (Takeuchi et al., 2011). *NEDD4L* mRNA is significantly expressed in Sézary syndrome (Booken et al., 2008). In addition, NEDD4L is expressed in melanoma but not in benign melanocytes and benign nevi tissue. NEDD4L overexpression promotes the growth of A2058 melanoma cells *in vivo*, whereas NEDD4L downregulation reduces the growth of G361 melanoma cells *in vitro* (Kito et al., 2014). Moreover, mRNA and protein levels of NEDD4L are much higher in the cytoplasm of invasive gallbladder cancer cells than in normal or dysplastic epithelial cells. Notably, the downregulation of NEDD4L cannot affect the growth of gallbladder cancer cells (Takeuchi et al., 2011).

### 2.3 Controversial NEDD4L expression

In addition to the above, NEDD4L expression is controversial in prostate cancer. Hu et al. (2009) reported that NEDD4L protein expression was downregulated in prostate cancer compared to levels in benign prostatic hyperplasia. In contrast, Hellwinkel et al. (2011) reported higher levels of *NEDD4L* mRNA in prostate cancer tissues than in adjacent normal tissues. Notably, three NEDD4L transcripts–NEDD4Lf, NEDD4Lg, and NEDD4Lh–were upregulated in prostate cancer cells in response to androgens (Qi et al., 2003; Sherk et al., 2008). NEDD4L was also downregulated in androgen-independent prostate cancer cells (Wang et al., 2018). These results suggest that dysregulation of NEDD4L may be related to androgen levels. Nonetheless, the role of NEDD4L in prostate cancer remains unclear, warranting further investigation.

### 2.4 Correlation in NEDD4L gene status, expression and prognosis

**FIGURE 3:**
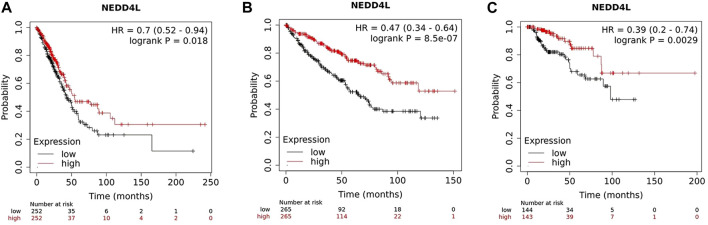
Survival plots depicting the good prognostic effect on OS of the higher expression of NEDD4L mRNA compared with the lower group in **(A)** LUAD, **(B)** kidney renal clear cell carcinoma, and **(C)** kidney renal papillary cell carcinoma.

So far, several studies and data analysis have shown that NEDD4L expression in tumors is closely associated with clinicopathological parameters and patient prognosis. In solid tumors, such as non-small-cell lung cancer (NSCLC) (Sakashita et al., 2013; Li G. et al., 2022), ovarian cancer (Yang et al., 2015), hepatocellular carcinoma (Zhao et al., 2018), renal clear cell carcinoma (Zhao et al., 2021), malignant glioma of the brain (He et al., 2012) and gastric cancer (Gao et al., 2012; Jiang et al., 2019), patients in the low-NEDD4L-expression group have worse prognoses and shorter survival than those in the high-expression group. Low NEDD4L expression is associated with larger tumor size, increased vascular invasion, lower differentiation, more lymph node and distant metastases, and progressive tumor stage (Sakashita et al., 2013; Yang et al., 2015; Li G. et al., 2022). NEDD4L is the only member of the NEDD4 family associated with overall survival in patients with LUAD (Li G. et al., 2022). Mutations in the NEDD4L gene also affect the prognosis of patients with NSCLC, and a bioinformatic analysis reported that in patients with NSCLC, the NEDD4L rs11660748 A>G and rs73440898 A>G adjusted overall survival hazard ratios were 1.31 and 1.27, respectively, implying that mutations in these two loci may affect patient prognosis (Yang S. et al., 2020). In AML, low NEDD4L expression is significantly associated with younger age at disease onset, higher leukocyte count, and higher numbers of naïve bone marrow/peripheral cells in patients (Chu et al., 2021). Moreover, multivariate analysis shows that NEDD4L expression is an independent prognostic factor in patients with gastric cancer (Gao et al., 2012; Jiang et al., 2019), squamous lung cancer (Sakashita et al., 2013) and glioma (He et al., 2012). Data from 7,489 patients in a public database (www.kmplot.com) shows the association between *NEDD4L* expression and survival. The samples are grouped according to the median expression of the *NEDD4L* gene. Kaplan–Meier plot shows that the high *NEDD4L* RNA expression correlated with longer overall survival (OS) in LUAD, kidney renal clear cell carcinoma, and kidney renal papillary cell carcinoma (Figure 3).

## 3 Upstream regulators of NEDD4L

NEDD4L has been shown to have a broad range of functions in a variety of tumors. Therefore, more studies focus on exploring the mechanisms of the upstream regulators. Relative mechanism includes non-coding RNA, phosphorylation, transcription regulations, etc. (Table 1).

**TABLE 1:** Upstream regulators modulate NEDD4L and its impact on cancers.

Mechanism	Cancer	Upstream regulators	Function	References
Regulation of NEDD4L by non-coding RNAs	Breast cancer	miR-675	Inhibits angiogenesis and metastasis	Guo et al. (2022a)
		miR-106b-25	Activates tumor-initiating cells	Guarnieri et al. (2018)
	Glioma	miR-10b- 5p	Promotes the proliferation, migration, and invasion	Li et al. (2022a)
		miR-513a-5p	Inhibits the sensitivity of glioma cells to temozolomide	Chen et al. (2019)
		miR-3679-5p	Enhances aerobic glycolysis and chemoresistance	Wang et al. (2020a)
	Lung cancer	miR-93	Enhances TGF-β-induced EMT	Qu et al. (2016)
	Colorectal carcinoma	circNElL3	Inhibits metastasis	Chen et al. (2023)
Phosphorylation of NEDD4L	Breast cancer	SGK1	Enhances the lung metastasis of breast cancer	Zhang et al. (2021e)
		Sek-1	Induces inactivation of NEDD4L	Hasna et al. (2018)
	Melanoma	MEK1/2	Inhibits melanoma cell multiplication and metastasis	Cui et al. (2020)
	Lung cancer	SGK1	Reduces the interaction of NEDD4L with GPX4	Cheng et al. (2023)
		AMPK	Inhibits lung Adenocarcinoma growth and metastasis	Ding et al. (2023)
Transcriptional regulation of NEDD4L	Ovarian cancer	DDB2	Inhibits cancer cell proliferation	Zhao et al. (2015)
	Lung cancer	EZH2	Contributes to progression and metastasis	Wang et al. (2019)
	Renal carcinoma	KSRP	Induces malignant progression	Yang et al. (2023)
Other mechanism	Lung cancer	ALCAP2	Inhibits cell proliferation, migration and invasion	Zhang et al. (2021c)
	Breast cancer	ESR1	Inhibits ionizing radiation-induced ferroptosis	Liu et al. (2022)
	Glioma	Paeoniflorin	Induces ferroptosis	Nie et al. (2022)
	Pancreatic cancer	NDRG1	Degrades oncogenic pSMAD2L	Kovacevic et al. (2013)

Molecules marked in red color are most recently discovered.

### 3.1 Regulation of NEDD4L by non-coding RNAs

Upstream signaling pathways that regulates NEDD4L expression have been recently identified. NEDD4L can be up- or downregulated by regulatory non-coding RNAs; however, the suppression of NEDD4L is predominant. Wang et al. found that miR-3679-5p, derived from M2 macrophages, enhanced aerobic glycolysis and chemoresistance in lung cancer by inhibiting the expression of the E3 ligase NEDD4L (Wang H. et al., 2020). Similarly, Qu et al. determined that miR-93 directly bound to the 3′-UTR of *NEDD4L* mRNA, resulting in the downregulation of NEDD4L expression at the protein level, ultimately promoting TGF-β-induced EMT in lung cancer cells (Qu et al., 2016). Long non-coding RNA H19 from the exosomes of M2 tumor-associated macrophages inhibits the interaction between ULK1 and its specific E3 ubiquitin ligase NEDD4L, stabilizing ULK1 expression and promoting autophagy in bladder cancer cells (Guo Y. et al., 2022). Moreover, in glioma cells, miR-10b-5p increases PIK3CA expression by downregulating NEDD4L expression, thereby promoting the proliferation, migration, and invasion of glioma cells by activating the PI3K-AKT pathway (Li G. et al., 2022). In addition, *NEDD4L* is a direct target of miR-513a-5p. It downregulates NEDD4L expression, ultimately reducing the sensitivity of glioma cells to temozolomide (Chen et al., 2019). Similarly, circNEIL3 recruits NEDD4L to ubiquitinate and downregulate YBX1, thereby inhibiting colorectal cancer metastasis (Chen et al., 2023). One study showed that circKDM4B sponged miR-675 to upregulate NEDD4L expression, thus inhibiting breast cancer progression (Guo X. Y. et al., 2022). Another study showed that NEDD4L could be directly inhibited by the miR-106b-25, which mediated breast cancer initiation by activating NOTCH1 signaling (Guarnieri et al., 2018). In AML, miR-10a is thought to be a microRNA that may directly target *NEDD4L* and is inversely correlated with NEDD4L expression (Chu et al., 2021).

### 3.2 Phosphorylation of NEDD4L

Protein phosphorylation is one of the most common and important post-translational modifications. NEDD4L was shown to be phosphorylated by several upstream regulators, including IκB kinase-β (IKKβ) (Edinger et al., 2009), AMP-activated kinase (AMPK) (Bhalla et al., 2006), and Akt1 (Lee et al., 2007). NEDD4L phosphorylation plays a key role in malignancy. AKT, a serine/threonine protein kinase that regulates cell growth and metabolism, is considered as a therapeutic target for tumors (Liao and Hung, 2010). Cui et al. reported that NEDD4L was phosphorylated by WT AKT at S342 and S448 (Salguero et al., 2022). Moreover, Zhang et al. reported that NEDD4L was phosphorylated by serum glucocorticoid-inducible kinase 1 (SGK1), leading to the release of Smad2 from inhibitory NEDD4L–Smad2 complexes, followed by the upregulation of connective tissue growth factor, ultimately promoting breast cancer metastasis (Liu et al., 2021). In addition, phosphorylation of NEDD4L is activated by MEK1/2 phosphorylation increased by JP1, a functional polypeptide, ultimately inhibiting melanoma proliferation and metastasis (Cui et al., 2020). In contrast, in ER^+^ breast cancer cells lacking Orai3 overexpression, Sek-1 phosphorylates NEDD4L, subsequently inducing its inactivation via sequestration of the 14-3-3 protein (Hasna et al., 2018). JAC4 inhibits epidermal growth factor receptor (EGFR)-driven LUAD growth and metastasis via the CTBP1-mediated JWA/AMPK/NEDD4L/EGFR axis. AMPK is able to stabilize NEDD4L expression by phosphorylating NEDD4L at Thr367 (Ding et al., 2023). These results indicate that NEDD4L can be activated or inactivated via phosphorylation.

### 3.3 Transcriptional regulation of NEDD4L

NEDD4L transcription is regulated by upstream regulators. Zhao et al. (2015) reported that DDB2 recruited the enhancer of zeste homolog 2 (EZH2) histone methyltransferase to repress NEDD4L transcription by enhancing histone H3 lysine 27 trimethylation (H3K27me3) at the NEDD4L promoter, which modulated TGF-β signal transduction in human ovarian cancer cells. In line with this finding, EZH2 inhibited the transcription of NEDD4L through H3K27 methylation, reducing NEDD4L ability to inhibit NSCLC cell proliferation, migration, and invasion (Wang et al., 2019). The N6-methyladenosine (m6A) modification is an mRNA methylation modification that promotes the stability of mRNA expression (Fu et al., 2014). Upregulation of FTO, an N6-methyladenosine (m6A) RNA demethylase, downregulates the m6A methylation level of *NEDD4L* mRNA, affecting its stability and leading to tumorigenesis (Cui et al., 2021). KH-type splicing regulatory protein (KHSRP), a versatile RNA-binding protein, aggravates the malignant progression of renal clear cell carcinoma by inhibiting the transcription or reducing post-transcriptional stability of NEDD4L (Yang et al., 2023).

### 3.4 Other mechanisms

Several upstream regulators are involved in either up- or downregulating NEDD4L. However, the specific mechanisms are unclear, and further research is required. β, β-dimethyl-acryl-alkannin (ALCAP2) upregulates NEDD4L expression, thereby enhancing β-catenin ubiquitination to inhibit the proliferation, migration, and invasion of LUAD cells (Liu et al., 2021). N-Myc downstream-regulated gene-1 (NDRG1), a tumor-suppressor gene, inversely correlates with tumor progression in a variety of tumors, including pancreatic cancer, and NDRG1 enhances the expression of NEDD4L in pancreatic cancer cells, which plays a role in degrading carcinogenic pSMAD2L (Kovacevic et al., 2013). Importantly, EGFR, often a ubiquitination substrate, downregulates NEDD4L, thereby enhancing mTOR signaling activity and promoting LUAD proliferation (Li G. et al., 2022). 14-3-3σ reportedly recruited S448-phosphorylation of NEDD4L to mediate ubiquitination and downregulation of hypoxia-inducible factor-1a (HIF-1a), thereby inhibiting colorectal cancer angiogenesis and enhancing sensitivity to bevacizumab (Liu et al., 2023).

## 4 Downstream substrates of NEDD4L

As an E3 ligase, most functions of NEDD4L were executed by regulating downstream substrates through ubiquitination, regulation of transcription, phosphorylation, etc. (Table 2). Notably, the regulation of downstream substrates by NEDD4L is not limited to downregulating their expression, but also maintaining substrate expression homeostasis or modulating the activity of downstream pathways.

**TABLE 2:** NEDD4L modulates substrates and its impact on cancers.

Mechanism	Cancer	Substrates	Function	References
Ubiquitination of substrates by NEDD4L	Breast cancer	PI3KCA	Inhibits angiogenesis and metastasis	Guo et al. (2022a)
		CD71	Inhibits ionizing radiation-induced ferroptosis	Liu et al. (2022)
		NOTCH1	Inhibits tumor-initiating cells	Guarnieri et al. (2018)
		CTR1	Inhibits PDK1-AKT Oncogenic Pathway	Guo et al. (2021)
		FOXO1	Inhibits breast cancer stemness	Yu et al. (2019)
		SLC7A11	Induces ferroptosis	Liu et al. (2021)
		P53	Induce resistance to chemotherapy	Hasna et al. (2018)
		YAP1	Inhibits autophagic cell death	Guo et al. (2023)
	Pancreatic cancer	ANXA2	Inhibits proliferation and metastasis	Wang et al. (2022)
		LTF	Inhibits ferroptosis	Wang et al. (2020b)
		ULK1	Downregulates autophagy and cell growth	Lee et al. (2020)
	Melanoma	SP1	Inhibits melanoma cell multiplication and metastasis	Cui et al. (2020)
	Glioma	PIK3CA.	Inhibits the proliferation, migration, and invasion	Li et al. (2022a)
		SphK2	Suppresses cell viability and invasion and promotes apoptosis	Wang et al. (2021)
		STAT3	Promotes ferroptosis	Nie et al. (2022)
	Lung cancer	β-catenin	Inhibits cell proliferation, migration and invasion	Zhang et al. (2021c)
		c-Myc	Inhibits aerobic glycolysis and chemoresistance	Wang et al. (2020a)
		UBE2T	Inhibits PI3K-AKT signaling	Chen et al. (2021)
		Notch2	Inhibits cell proliferation	Lin et al. (2022)
		CPNE1	Inhibits proliferation and metastasis	Zhang et al. (2021b)
		PD-L1	Enhances anti-tumor immune response	Zhong et al. (2022)
		GCN2	Inhibits apoptosis	Wei et al. (2015)
		EGFR	Inhibits lung adenocarcinoma growth and metastasis	Cheng et al. (2023)
		GPX4	Promotes ferroptosis and chemosensitivity	Cheng et al. (2023)
	Gastric cancer	RDX	Inhibits gastric cancer tumorigenicity and metastasis	Tang et al. (2023)
	Bladder cancer	ULK1	Inhibits autophagy	Guo et al. (2022b)
	Prostate cancer	PHF8	Represses proliferation	Feng et al. (2023)
	Esophageal carcinoma	c-Myc	Inhibits cycle progression, and glutamine metabolism	Cheng et al. (2022)
	Colorectal cancer	STK35	Inhibits glycolysis and enhance chemosensitivity	Yang et al. (2020a)
		Wnt3	Regulates gut microbiota	Wang et al. (2023)
		YBX1	Inhibits tumor metastasis	Chen et al. (2023)
	Oral carcinoma	ENO1	Inhibits glycolysis and proliferation	Zhang et al. (2022)
Phosphorylation regulation of substrates by NEDD4L	Hepatocellular carcinoma	ERK1/2	Mediates apoptosis	Zhao et al. (2018)
Transcriptional regulation of substrates by NEDD4L	Gallbladder cancer	MMP-1; MMP-13	Increases the invasive activity	Takeuchi et al. (2011)
Other mechanisms	Multiple myeloma	19S proteasome	Promotes autophagy and bortezomib sensitivity	Huang et al. (2022)
	Pancreatic cancer	ASCT2	Downregulates autophagy and cell growth	Lee et al. (2020)
	Bladder cancer	p62	Inhibits migration, invasion, and cisplatin resistance	Wu et al. (2023)

Molecules marked in red color are most recently discovered.

### 4.1 Ubiquitination of substrates by NEDD4L

As a HECT-type E3 ubiquitin ligase, NEDD4L regulates substrates mainly through ubiquitination. Tumor-associated substrates of NEDD4L have been identified in numerous pathway proteins and membrane receptors.

Class IA phosphoinositide 3-kinases (PI3Ks) are lipid kinases that integrate signals from growth factors and hormones and play a major role in PI3K-associated cancer progression (Cantley, 2002). NEDD4L has been identified as an E3 ligase that mediates the ubiquitination and downregulation of PIK3CA, the catalytic subunit of class IA PI3Ks, thereby inhibiting the PI3K-AKT signaling pathway and VEGFA secretion in breast cancer (Kok et al., 2009; Wang et al., 2016; Guo X. Y. et al., 2022). Moreover, this modification also inhibits glioma progression induced by M2 polarization of macrophages in gliomas (Li B. et al., 2022). Ubiquitin-conjugating enzyme E2T (UBE2T), an oncogene, is an upstream molecule in PI3K-AKT signaling (Yin et al., 2020). NEDD4L inhibits PI3K-AKT signaling by targeting the ubiquitination and degradation of UBE2T (Chen et al., 2021). Additionally, Unc51-like autophagy activating kinase 1 (ULK1) is a serine/threonine kinase that initiates autophagy in mammals (Jiang et al., 2015; Chen et al., 2023). NEDD4L binds to ULK1 in pancreatic cancer cells and is involved in the ubiquitination and subsequent degradation of ULK1 to downregulate autophagy and mitochondrial metabolism, ultimately suppressing the growth and survival of pancreatic cancer cells (Lee et al., 2020). In bladder cancer, long non-coding RNA H19 from the exosomes of M2 tumor-associated macrophages interfers K48-linked polyubiquitination of ULK1 mediated by NEDD4L, stabilizing ULK1 expression and promoting bladder cancer cell autophagy (Guo Y. et al., 2022). The NOTCH pathway is highly conserved, and aberrant NOTCH signaling is closely associated with tumorigenesis (Meisel et al., 2020). The NOTCH1 and NOTCH2 proteins are downregulated by the NEDD4L-mediated ubiquitin–proteasome system. NOTCH1 and NOTCH2 downregulation inhibits the proliferation of tumor-initiating cells in several breast cancer cell lines and LUAD cells, respectively (Guarnieri et al., 2018; Lin et al., 2022). The Myc family plays a central role in almost every aspect of the oncogenic process by coordinating tumor cell proliferation, apoptosis, differentiation, and metabolism (Dang, 2012; Chen et al., 2018). NEDD4L mediates the ubiquitination and degradation of c-Myc, a Myc family member, to inhibit cell viability, cell cycle progression, and glutamine metabolism in esophageal lymphoid carcinoma, as well as cellular glycolysis and chemoresistance in lung cancer (Wang H. et al., 2020; Cheng et al., 2022). Abnormal activation of the Wnt/β-catenin signaling pathway leads to abnormal cell proliferation, inhibition of apoptosis, EMT, immune escape, and other carcinogenic cellular behaviors (Pan et al., 2018; Flanagan et al., 2019; Luke et al., 2019). One study reported that NEDD4L might exert a tumor-suppressive effect in colorectal cancer via Wnt signaling suppression (Tanksley et al., 2013). Subsequently, it was reported that both Wnt and β-catenin could be downregulated by NEDD4L via ubiquitination. Downregulation of Wnt3 negatively regulated the progression of colorectal cancer, but this effect was diminished by the (pro)renin receptor, and downregulation of β-catenin inhibited the transcription of Wnt-triggered genes, such as the survival hormones cyclin D1 and MMP9 (Tanksley et al., 2013; Wang et al., 2023). NEDD4L also enhanced the sensitivity of glioma cells to temozolomide by inhibiting Wnt/β-catenin signaling (Chen et al., 2019). The Orai3 protein, a highly selective calcium channel, plays a key role in calcium entry. In Orai3-overexpressing breast cancer cells, NEDD4L ubiquitinates the P53 protein and induces its degradation (Hasna et al., 2018). Y-box-binding protein 1 (YBX1; also known as YB-1) plays a crucial role in tumor metastasis in colorectal cancer (Evdokimova et al., 2009; El-Naggar et al., 2019; Ruan et al., 2020), and NEDD4L mediates YBX1 ubiquitination and degradation (Chen et al., 2023). High expression of SLC7A11, a critical regulator of ferroptosis, predicts poor prognosis in breast cancer (Xu et al., 2020; Yang et al., 2021). After exposure of breast cancer cells to ionizing radiation, interactions between NEDD4L and SLC7A11 increase, followed by SLC7A11 ubiquitination and degradation, ultimately inducing ferroptosis in breast cancer cells (Liu et al., 2021). SP1 is identified as a substrate of NEDD4L, and NEDD4L downregulates it via ubiquitination at K685 site, inhibiting the proliferation and metastasis of melanoma mediated by the SP1-integrin αvβ3 pathway (Cui et al., 2020). Serine/threonine kinase 35 (STK35) plays a pivotal role in regulating the cell cycle, and abnormal levels are associated with various tumors. STK35 is ubiquitinated by NEDD4L and promotes glycolysis by regulating the AKT signaling pathway to affect colorectal cancer chemotherapy resistance (Yang H. et al., 2020). Ubiquitination of the tumor oncogene sphingosine kinase 2 (SphK2) mediated by NEDD4L overexpression inhibits glioma cell viability and invasion as well as promotes glioma cell apoptosis (Wang et al., 2021). Alpha-enolase (ENO1), an enzyme that catalyzes glycolysis, is highly expressed in oral squamous cell carcinoma. ENO1 is a substrate of NEDD4L and is downregulated via ubiquitination to inhibit glycolysis and cancer cell proliferation (Yin et al., 2018; Zhang et al., 2022). The C-terminus of forkhead box O1 (FOXO1) is downregulated by NEDD4L via ubiquitination at K463 site, which inhibits breast cancer stem cells (Yu et al., 2019). Copine-1 (CPNE1) expression has been positively correlated with NSCLC development, TNM stage, lymphatic metastasis, and distant metastasis, and NEDD4L mediates CPNE1 ubiquitination at K157 residue to promote its degradation (Liu et al., 2021). Ubiquitination of STAT3 by NEDD4L induces its downregulation in glioma cells (Nie et al., 2022). General control non-depressible kinase 2 (GCN2) is a promising target for cancer therapy. In A549 lung cancer cells, NEDD4L mediates the ubiquitination and downregulation of GCN2, inhibiting the GCN2-induced apoptosis of cancer cells (Wei et al., 2015). Glutathione peroxidase 4 (GPX4) determines the sensitivity of lung cancer cells to etoposide-induced ferroptosis, and NEDD4L can ubiquitinate GPX4, resulting in its subsequent degradation (Cheng et al., 2023). Cytoplasmic yes-associated protein 1 (YAP1) promotes autophagic death in breast cancer cells, and NEDD4L mediates ubiquitination and downregulation of YAP1 (Guo et al., 2023). Human organic anion transporter 3 (hOAT3) is highly expressed in the kidneys and plays a key role in the secretion of clinically important drugs, including anti-cancer drugs (You, 2002; Erdman et al., 2006). NEDD4L downregulates hOAT3 expression by enhancing hOAT3 ubiquitination (Zhang et al., 2018). Activated Cdc42-associated kinase1 (Ack1), a non-receptor tyrosine kinase with a unique structure, is closely associated with the biological behavior of malignant tumors (Prieto-Echagüe et al., 2010). NEDD4L may downregulate Ack1 via ubiquitination, thereby regulating EGFR activity (Chan et al., 2009). Intercellular adhesion molecule 2 (ICAM2), a transmembrane glycoprotein, can promote the NEDD4L-mediated ubiquitination and degradation of RDX, thereby inhibiting the tumorigenicity and metastasis of gastric cancer (Tang et al., 2023). HIF-1a is ubiquitinated and degraded by NEDD4L to inhibit colorectal cancer angiogenesis and enhance sensitivity to bevacizumab (Liu et al., 2023).

Ubiquitination mediated by NEDD4L also plays a role in regulating its member receptors. CD71, a transferrin receptor, is an important mediator of ferroptosis, and its degradation, mediated by NEDD4L via the ubiquitin–proteasome system, inhibits ferroptosis in breast cancer cells after exposure to ionizing radiation (Candelaria et al., 2021; Liu et al., 2022). Cu activates the PDK1-AKT oncogenic pathway via copper transporter 1 (CTR1) to promote tumorigenesis. CTR1 is abnormally elevated in breast cancer, and NEDD4L downregulates CTR1 via ubiquitination to inhibit the PDK1-AKT pathway (Guo et al., 2021). PD-L1 is shown to be a substrate for NEDD4L, which inhibits PD-L1 levels via ubiquitination to enhance the anti-tumor immune response of NSCLC (Zhong et al., 2022). The intestinal stem cell marker LGR5 was also found to be a substrate for NEDD4L, which can downregulate it via the ubiquitin–proteasome or lysosomal system to negatively regulate Wnt/β-catenin signaling (Barker et al., 2007; Novellasdemunt et al., 2020). Abnormal activation of EGFR is an important driver of human cancer (Arteaga and Engelman, 2014). The WW domain of the E3 ubiquitin ligase NEDD4L interacts with EGFR at its K716, thereby promoting its ubiquitination at K716 and degradation in LUAD (Ding et al., 2023).

### 4.2 Phosphorylation and transcriptional regulation of substrates by NEDD4L

Moreover, NEDD4L exerts its biological functions by phosphorylating substrates and regulating substrate transcription. A previous study reported that NEDD4L might inhibit cell proliferation and promote apoptosis by phosphorylating ERK1/2 (Zhao et al., 2018). In addition, NEDD4L overexpression may lead to gallbladder cancer invasion by decreasing the transcription of *MMP1* and *MMP13* (Takeuchi et al., 2011).

### 4.3 Other mechanisms

Additional substrates and downstream signaling pathways are regulated by NEDD4L. However, the exact mechanisms are unclear. Phosphorylated Smad2/3 can be targeted by NEDD4L for protein degradation. Subsequently, pSmad2/3-dependent TGF-β signaling is inhibited in lung cancer (Qu et al., 2016). The mTOR signaling pathway is closely associated with tumor proliferation (Morita et al., 2015). In LAUD, p-mTOR is targeted for degradation by NEDD4L, which inhibits the mTOR signaling pathway (Liu et al., 2022). Oncogenic pSMAD2 is downregulated by NEDD4L overexpression (Kovacevic et al., 2013). Lactotransferrin (LTF) is a protein that binds directly to NEDD4L, and NEDD4L-targeted degradation of LTF inhibits iron accumulation and subsequent iron-related death in various cancer cells (Wang H. et al., 2020). NEDD4L can also bind to the 19S proteasome, inhibiting its hydrolytic function and enhancing autophagy and sensitivity to bortezomib in multiple myeloma cells (Huang et al., 2022). The p62/Keap1/nuclear factor erythroid 2-related factor 2 (NRF2) pathway exerts oncogenic effects in bladder cancer (Li et al., 2020). NEDD4L also inhibits the Keap1/NRF2 pathway by suppressing p62 expression (Wu et al., 2023).

## 5 NEDD4L functions in cancer

Evidences show the implicated mechanism of NEDD4L in both solid and non-solid tumors, which is involved in a range of pathophysiological processes, indicating that NEDD4L plays an anti-tumor effect in variety aspects referring tumor microenvironment and metabolism. Whereas it acts as a pro-tumor factor as well. These results lead us to a deeper understanding and awareness of it and provide a new direction of targeting drug strategies.

### 5.1 Regulation of cell proliferation

Since some substrates of NEDD4L are key factors in the signaling pathways that regulate tumor cell proliferation, the downregulation of these substrates by NEDD4L targeting might inhibit cell proliferation.

NEDD4L mediates the ubiquitination and degradation of β-catenin, NOTCH2, CPNE1, and PD-L1, resulting in their downregulation and thereby inhibiting NSCLC cell proliferation (Zhang R. et al., 2021; Liu et al., 2021; Lin et al., 2022; Zhong et al., 2022). NEDD4L ubiquitinates SP1, leading to its degradation, which inhibits melanoma cell proliferation (Cui et al., 2020). Downregulation of NEDD4L increases PIK3CA expression, thereby promoting glioma cell proliferation by activating the PI3K/AKT pathway (Li G. et al., 2022). NEDD4L inhibits the proliferation of prostate cancer cells by degrading plant homeodomain finger protein 8 (PHF8/KDM7B) through the ubiquitin–proteasome system (Feng et al., 2023). NEDD4L inhibits the proliferation of oral squamous cells by inducing the ubiquitination and degradation of ENO1 (Zhang et al., 2022). NEDD4L has anti-proliferative effects on the leukemia cell line K562 (Chu et al., 2021). LINC00941 interacts with ANXA2 and inhibits NEDD4L-mediated ANXA2 degradation to promote cell proliferation in pancreatic cancer (Wang et al., 2022). In hepatocellular carcinoma (HCC), NEDD4L inhibits cell proliferation via the MAPK/ERK pathway (Zhao et al., 2018). In addition, one study showed that the loss of NEDD4L promoted the proliferation of intestinal stem cells (Novellasdemunt et al., 2020). However, some upstream molecules of NEDD4L might promote cell proliferation by inhibiting NEDD4L expression. NEDD4L, which is transcriptionally repressed by DDB2, enhances TGF-β signaling in human ovarian cancer cells, ultimately inhibiting ovarian cancer cell proliferation (Zhao et al., 2015). EZH2 reduces the ability of NEDD4L to inhibit NSCLC cell proliferation by inhibiting NEDD4L transcription (Wang et al., 2019). These results suggest that NEDD4L inhibits or promotes cancer cell proliferation.

### 5.2 Regulation of cell apoptosis

Several studies have reported that NEDD4L promoted apoptosis in tumor cells by downregulating downstream pathways. NEDD4L can downregulate STK35 via ubiquitination, resulting in apoptosis in colorectal cancer cells (Yang H. et al., 2020). Moreover, NEDD4L-mediated ubiquitination of SphK2 promotes glioma cell apoptosis (Wang et al., 2021). Additionally, NEDD4L induces apoptosis in hepatoma and leukemia cells (Zhao et al., 2018; Chu et al., 2021). NEDD4L also inactivates the p62/Keap1/Nrf2 pathway to promote bladder cancer cell apoptosis (Wu et al., 2023). However, NEDD4L reportedly mediated GCN2 ubiquitination and subsequent proteasomal degradation, which prohibited cancer cell apoptosis (Wei et al., 2015).

### 5.3 Regulation of ferroptosis

Ferroptosis is non-apoptotic regulatory cell death caused by iron accumulation and subsequent lipid peroxidation and has great potential in tumor treatment (Zhao et al., 2022). One study showed that NEDD4L-mediated degradation of LTF inhibited intracellular iron accumulation and subsequent oxidative damage-mediated ferroptosis in various cancer cells (Wang Y. et al., 2020). Another study reported that ESR1 enhanced the binding of NEDD4L to CD71, promoted CD71 ubiquitination and degradation, and inhibited ionizing radiation-induced ferroptosis in breast cancer cells (Liu et al., 2022). These two studies confirmed that NEDD4L negatively affected ferroptosis. Conversely, several studies have reported a positive role of NEDD4L in ferroptosis. Liu et al. reported that the interaction between NEDD4L and SLC7A11 was enhanced after ionizing radiation in breast cancer cells, followed by the ubiquitination and degradation of SLC7A11, ultimately triggering ferroptosis (Gao et al., 2021). In addition, paeoniflorin induces ferroptosis in human glioma cells by increasing NEDD4L-dependent STAT3 ubiquitination (Li G. et al., 2022). GPX4 enhances ferroptosis resistance in NSCLC cells (Yang et al., 2014). NEDD4L is thought to mediate the ubiquitination and degradation of GPX4 to facilitate ferroptosis (Cheng et al., 2023). However, lactic acid drives the activation of the p38-SGK1 pathway, attenuating interactions between NEDD4L and GPX4, as well as the subsequent ubiquitination and degradation of GPX4 to confer ferroptosis resistance in NSCLC (Cheng et al., 2023). Therefore, NEDD4L exerts a dual regulatory effect on ferroptosis.

### 5.4 Regulation of the cell cycle

Cyclin D1 is an important regulator of the cell cycle that plays a role in the uncontrolled proliferation of tumor cells (Kim and Diehl, 2009; Montalto and De Amicis, 2020). NEDD4L upregulation inhibits the nuclear translocation of β-catenin and facilitates the binding of ubiquitin to β-catenin, ultimately affecting cyclin D1 transcription (Zhang W. et al., 2021). In oral squamous cell carcinoma, NEDD4L overexpression results in an increase in the number of G0/G1 cells and a decrease in the number of S-phase cells, indicating that NEDD4L triggers cell cycle arrest. However, ENO1 overexpression reverses this effect (Zhang et al., 2022). Moreover, NEDD4L may inhibit the proliferation of LUAD cells by inducing S-phase cell cycle arrest (Li G. et al., 2022). Therefore, NEDD4L may inhibit tumor growth by regulating the cell cycle.

### 5.5 Regulation of cell migration and invasion

NEDD4L mediates the ubiquitination and downregulation of PI3KCA, inhibiting breast cancer and glioma cell migration and invasion *in vitro* (Guo X. Y. et al., 2022; Li B. et al., 2022). Moreover, NEDD4L-mediated ubiquitination of SphK2 inhibits glioma cell survival and invasion (Wang et al., 2021). One study confirmed that ALCAP2-mediated upregulation of NEDD4L inhibits the migration and invasion of LUAD cells (Zhang W. et al., 2021). Similarly, another study reported that inhibition of NEDD4L transcription reduced its ability to inhibit the migration and invasion of NSCLC cells (Wang et al., 2019). Additionally, NEDD4L inactivates the p62/Keap1/Nrf2 pathway, inhibiting bladder cancer cell migration and invasion (Wu et al., 2023). Notably, Takeuchi et al. (2011) reported that silencing of the *NEDD4L* gene reduced the invasion activity of cultured gallbladder cancer cells without affecting cell growth. LINC00941 accelerates cell migration and invasion by suppressing NEDD4L-mediated ANXA2 degradation (Wang et al., 2022). Thus, NEDD4L mainly inhibits the migration and invasion of human tumor cells.

### 5.6 Regulation of EMT

EMT is the process by which epithelial cells acquire a mesenchymal phenotype, allowing them to detach from the primary tumor and metastasize to distant sites (Cho et al., 2019). EMT is closely related to malignant tumor progression and is an important mechanism for tumor invasion, metastasis, and the development of drug resistance (Thiery et al., 2009; Cho et al., 2019). Transforming growth factor-β (TGF-β) induces EMT (Robinson and Sandler, 2013). Downregulation of NEDD4L promotes TGF-β-induced EMT, leading to lung cancer metastasis (Qu et al., 2016). SphK2 expression is upregulated in glioma tissues and promotes EMT in glioma cells via the AKT/β-catenin pathway (Wang et al., 2021). Upregulation of NEDD4L expression mediates SphK2 ubiquitination and downregulation, implying that NEDD4L may inhibit SphK2-induced EMT (Wang et al., 2021). However, whether NEDD4L is directly involved in the inhibition of EMT in tumor cells warrants further study.

### 5.7 Regulation of drug sensitivity

NEDD4L plays a crucial role in drug sensitivity during cancer therapy. One report showed that NEDD4L regulated the AKT signaling pathway by ubiquitinating STK35, ultimately affecting chemoresistance in colorectal cancer (Yang H. et al., 2020). Another study reported that IGF-1-enhanced miR-513a-5p reduced the sensitivity of glioma cells to temozolomide by targeting the NEDD4L-downregulated Wnt/β catenin pathway (Chen et al., 2019). Moreover, the downregulation of NEDD4L mediated by miR-3679-5p stabilizes c-Myc, thereby enhancing cisplatin resistance in lung cancer cells (Wang H. et al., 2020). In addition, NEDD4L inhibits cisplatin resistance in bladder cancer cells by inactivating the p62/Keap1/Nrf2 pathway (Wu et al., 2023). Huang et al. reported that suppression of NEDD4L expression diminished the sensitivity of multiple myeloma to bortezomib, which was mainly mediated by low NEDD4L expression via autophagy inhibition (Huang et al., 2022). In Orai3-overexpressing breast cancer cells, NEDD4L mediates the ubiquitination and degradation of its target proteins (including either p53 regulators or p53), inducing chemotherapy resistance (Hasna et al., 2018). Further studies are required to confirm the involvement of NEDD4L in regulating the sensitivity of malignancies to therapeutic agents.

### 5.8 Regulation of tumor metabolism

Metabolism is a fundamental process for all cells. The metabolism of cancer cells plays a key role in the survival and growth of cancer cells (Li and Le, 2018). NEDD4L has been extensively reported to regulate glucose metabolism and glutamine metabolism. Wang H. et al. (2020) reported that macrophage-derived miR-3679-5p stabilized c-Myc by inhibiting NEDD4L, enhancing aerobic glycolysis and chemotherapy resistance in lung cancer. Likewise, Yang H. et al. (2020) demonstrated that NEDD4L could ubiquitinate STK35 to downregulate STK35-promoted glycolysis and chemotherapy resistance in colorectal cancer. Moreover, NEDD4L mediates the ubiquitination and downregulation of ENO1, thereby inhibiting glycolysis in oral squamous cell carcinoma (Zhang et al., 2022). Another study reported that NEDD4L could inhibit glutamine metabolism in ESCC via c-Myc ubiquitination to reduce GLS1 and SLC1A5 expression (Cheng et al., 2022). Moreover, NEDD4L can reportedly reduce the level of ASCT2, a glutamine transporter, to inhibit autophagy and mitochondrial metabolism in pancreatic cancer (Lee et al., 2020).

### 5.9 Regulation of tumor microenvironment

Tumor microenvironment (TME) refers to the surrounding microenvironment in which tumor cells exist, including surrounding blood vessels, immune cells, fibroblasts, myeloid inflammatory cells, various signaling molecules and extracellular matrix (ECM) (Xiao and Yu, 2021). The role of NEDD4L in the TME has also been reported. In NSCLC, NEDD4L can mediate the ubiquitination and downregulation of PD-L1, increasing the proportion of CD8^+^ T cells and the content of IL-2 and INF-γ (Zhong et al., 2022). NEDD4L can also mediate the ubiquitination and downregulation of EGFR to inhibit the growth and metastasis of lung adenocarcinoma (Ding et al., 2023). In breast cancer, miR-675-upregulated NEDD4L can catalyze the ubiquitination of PI3KCA, thereby inhibiting VEGFA secretion and ultimately inhibiting blood vessel formation (Guo X. Y. et al., 2022). In addition, the downregulation of NEDD4L can promote M2 macrophage polarization, leading to tumor progression in glioma (Li B. et al., 2022).

### 5.10 The oncogenic role of NEDD4L in cancer

Although NEDD4L has been widely reported to exert cancer-suppressing effects, several studies have elucidated its carcinogenic role. For instance, NEDD4L might be involved in the development of CD5^+^, relapsed, and refractory diffuse large B-cell lymphoma (Qu et al., 2019). DDB2 enhances TGF-β signal transduction by downregulating NEDD4L transcription to inhibit ovarian cancer cell proliferation (Zhao et al., 2015). In Orai3-overexpressing breast cancer cells, NEDD4L mediates the ubiquitination and degradation of its target proteins (including either p53 regulators or p53), inducing resistance to chemotherapy (Hasna et al., 2018). Moreover, the upregulation of NEDD4L enhances the transcription of *MMP1* and *MMP13*, leading to aggressive gallbladder cancer (Takeuchi et al., 2011). GCN2, CD71, and LTF are substrates of NEDD4L, which exerts carcinogenic effects by downregulating them (Wei et al., 2015; Wang H. et al., 2020; Liu et al., 2022). In addition, one study reported that NEDD4L level was higher in prostate cancer tissues than in adjacent normal tissues, and NEDD4L might contribute to the development of prostate cancer by inhibiting the TGF-β signaling pathway (Hellwinkel et al., 2011). Another study reported that NEDD4L downregulation inhibited the growth of G361 melanoma cells cultured *in vitro*, while NEDD4L expression promoted the growth of A2058 melanoma cells *in vivo* (Kito et al., 2014). These two studies contradict the findings of other studies on the role of NEDD4L in prostate cancer and melanoma.

## 6 Conclusion and perspectives

**FIGURE 4:**
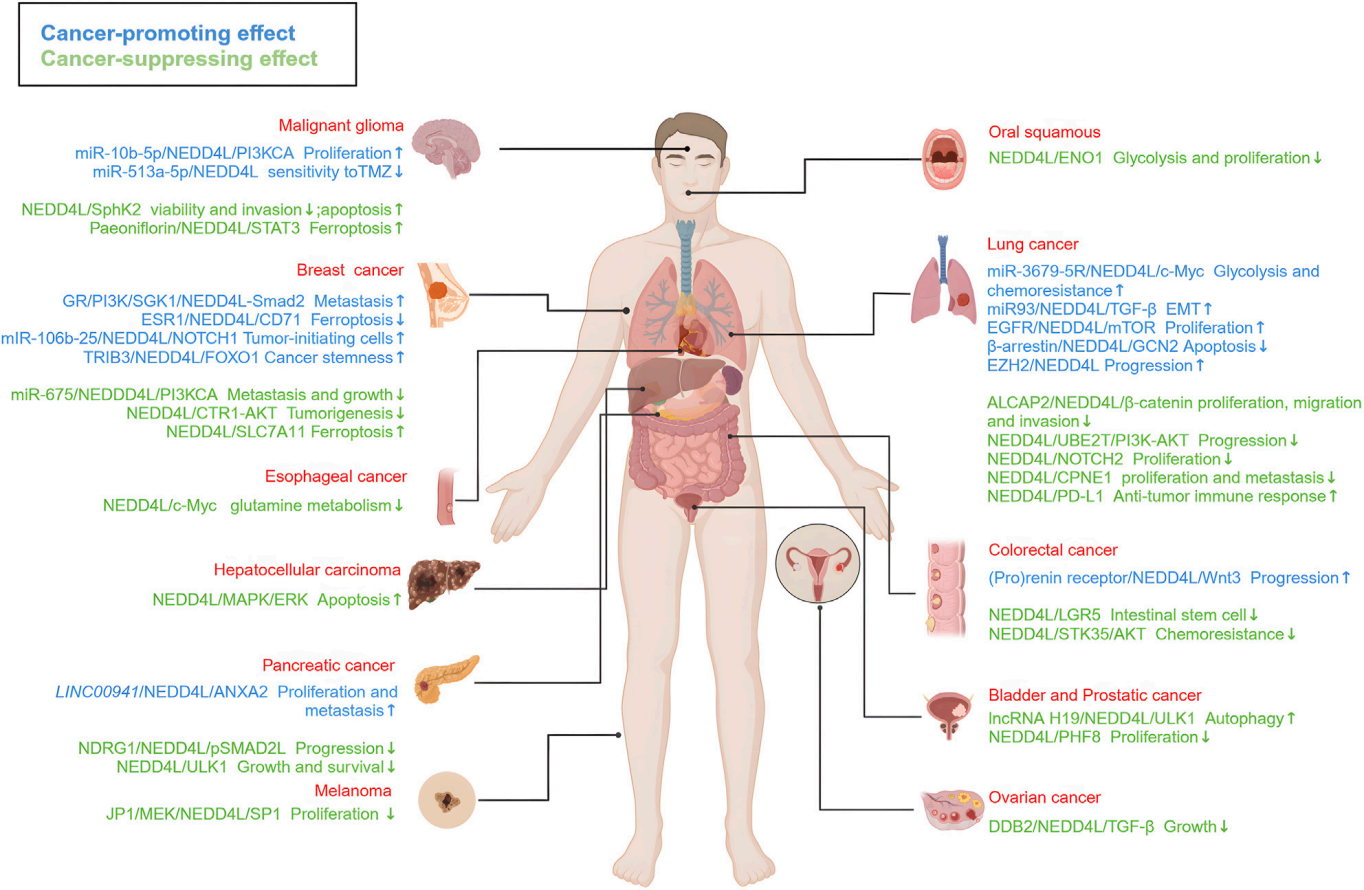
The roles of NEDD4L in different types of tumors. NEDD4L is highly associated with a range of malignancies, including glioma, oral cancer, lung cancer, breast cancer, liver cancer, esophageal cancer, colorectal cancer, pancreatic cancer, ovarian cancer, bladder cancer, prostate cancer, and melanoma. The signaling pathways associated with tumor-suppressor activity (marked in blue) and tumor-promoter activity (marked in green). These signaling pathways regulate the growth, proliferation, migration and invasion, apoptosis, autophagy, EMT, and drug sensitivity of tumor cells, as well as tumorigenesis and tumor progression. https://biorender.com.

Overall, NEDD4L plays a dual role in different types of tumors (Figure 4). NEDD4L involved in tumorigenesis, progression and metastasis is well-established. In particular, it has an effect on the tumor immune microenvironment and other pathophysiological processes, such as cell autophagy, cell cycle regulation and tumor metabolism. Though, the main mechanisms have not been elucidated. Potential reasons for this controversial effect include tissue-specific expression as well as a broad spectrum of ubiquitin substrates.

Meanwhile, NEDD4L regulates downstream protein expression, especially membrane protein in dynamic equilibrium. Besides, it also participates in protein transportation and maintenance of cytoplasmic protein homeostasis, instead of degradation of substrates alone. Therefore, to explore the features of NEDD4L in tumors, we should not only focus on the expression level of the substrates, but also on the function of its regulatory networks. Additionally, more detail studies are still needed, especially in modification site and degradation mechanism of the substrates.

We also believe that anti-tumor strategies targeting NEDD4L should be better concentrated on the breakdown of the association between NEDD4L and its substrates, rather than adjusting the activity of NEDD4L itself, based on higher specificity, which is the cornerstone of pharmaceutic. For example, proteolysis targeting chimera (PROTACT) drug technique chooses the RING E3 ligase as a ligand-selective component to target the degradation of non-drug-eligible target proteins with no binding pocket. Furthermore, commitments to incorporate HECT-like E3 ligases into PROTACT drug preparation will regulate additional proteins expression and pathophysiological processes. Thus, we conclude the dual functions and controversial mechanism of NEDD4L to provide more directional references and thinking perspectives for future researches.
